# Peptides derived from hookworm anti-inflammatory proteins suppress inducible colitis in mice and inflammatory cytokine production by human cells

**DOI:** 10.3389/fmed.2022.934852

**Published:** 2022-09-09

**Authors:** Claudia Cobos, Paramjit S. Bansal, David T. Wilson, Linda Jones, Guangzu Zhao, Matthew A. Field, Ramon M. Eichenberger, Darren A. Pickering, Rachael Y. M. Ryan, Champa N. Ratnatunga, John J. Miles, Roland Ruscher, Paul R. Giacomin, Severine Navarro, Alex Loukas, Norelle L. Daly

**Affiliations:** ^1^Centre for Molecular Therapeutics, Australian Institute of Tropical Health and Medicine, James Cook University, Cairns, QLD, Australia; ^2^Centre for Tropical Bioinformatics and Molecular Biology, Australian Institute of Tropical Health and Medicine, James Cook University, Cairns, QLD, Australia; ^3^Garvan Institute of Medical Research, Darlinghurst, NSW, Australia; ^4^Infection and Inflammation Program, QIMR Berghofer Medical Research Institute, Brisbane, QLD, Australia; ^5^Faculty of Health, Woolworths Centre for Childhood Nutrition Research, Queensland University of Technology, South Brisbane, QLD, Australia

**Keywords:** protein structure, nuclear magnetic resonance, peptide synthesis, colitis mouse model, inflammation

## Abstract

A decline in the prevalence of parasites such as hookworms appears to be correlated with the rise in non-communicable inflammatory conditions in people from high- and middle-income countries. This correlation has led to studies that have identified proteins produced by hookworms that can suppress inflammatory bowel disease (IBD) and asthma in animal models. Hookworms secrete a family of abundant netrin-domain containing proteins referred to as AIPs (Anti-Inflammatory Proteins), but there is no information on the structure-function relationships. Here we have applied a downsizing approach to the hookworm AIPs to derive peptides of 20 residues or less, some of which display anti-inflammatory effects when co-cultured with human peripheral blood mononuclear cells and oral therapeutic activity in a chemically induced mouse model of acute colitis. Our results indicate that a conserved helical region is responsible, at least in part, for the anti-inflammatory effects. This helical region has potential in the design of improved leads for treating IBD and possibly other inflammatory conditions.

## Introduction

The rise in incidence of autoimmune and related inflammatory conditions in high- and middle-income countries appears to be correlated with a decrease in the prevalence of helminths such as hookworms, potentially due to the immune-modifying abilities of these pathogens (1–3). This correlation has led to studies of the therapeutic or prophylactic efficacy of parasite excretory/secretory (ES) proteins in autoimmune conditions such as inflammatory bowel disease (IBD) and asthma (4, 5). IBDs affect the gastrointestinal tract and can be sub-classified into two main conditions: ulcerative colitis (UC) and Crohn’s disease (CD) (6). The causes of IBD are still unknown, but a combination of dysregulated immune responses and environmental factors appear to be involved (7). Asthma primarily affects the respiratory tract, and its incidence is rising in industrialized and developing countries, generating a burden on their health services (8). Current treatments for both IBD and asthma have significant limitations (9, 10), and consequently, new drugs are required.

The excretory/secretory (ES) products of hookworms are a diverse source of molecules with potential in the treatment of inflammatory diseases such as IBD and asthma (5, 11–13). The ES products comprise a complex mixture of extracellular vesicles, proteins, carbohydrates, small molecules, metabolites and lipids secreted by the parasite (14). Proteomic profiling of ES products of the dog hookworm, *Ancylostoma caninum*, indicated the presence of 250 different proteins (14, 15). Several of these proteins have sequence homology to a family of mammalian proteins known as tissue inhibitors of matrix-metalloproteinases (TIMPs), including *Ac*-TMP-1 and *Ac*-TMP-2 (16). Despite sequence and predicted structural similarity to TIMPs, these hookworm proteins do not seem to possess metalloprotease inhibitory activity, and their netrin domains likely perform unrelated functions (16). *Ac*-TMP-1 and *Ac*-TMP-2 have subsequently been referred to as *Ac*-AIP (Anti-Inflammatory Protein)-1 and *Ac*-AIP-2, and have been tested in mouse models of colitis (17) and asthma (5), respectively. Recombinant forms of both proteins significantly alleviate the disease symptoms in these models, reduce immunopathology and suppress the expression of inflammatory cytokines in treated animals and in human immune cells *ex vivo*. *Na*-AIP-1 is a related protein from the human hookworm, *Necator americanus* which has also recently been shown to display anti-inflammatory activity and protect against T cell transfer colitis in mice (4). The biological targets of these proteins are unknown, and there is no information available on the structure-function relationships.

Analysis of three-dimensional protein structures can provide clues to regions important in activity, and insight into the process of “downsizing” proteins (18–20). The downsizing process can be valuable for the design of peptide-based drug leads that have lower immunogenicity, greater tissue penetration, and are cheaper to manufacture than larger proteins (21, 22). We have previously used a structure-based downsizing approach in the development of wound healing peptides (23, 24). Here we have applied this approach to selected hookworm TIMP-like proteins from three different species. We have focused on conserved elements of secondary structure that are solvent accessible, with the aim of developing peptides with potential in treating colitis.

The three hookworm AIP proteins chosen for this study are *Ace*ES-2 from *Ancylostoma ceylanicum, Ac*-AIP-2 from *A. caninum*, and *Na*-AIP-1 from *N. americanus*. Although there is no bioactivity data associated with *Ace*ES-2, it was included in our study because a crystal structure is available (25). Our design studies were based on this crystal structure, as well as and modeled structures of *Ac*-AIP-2 and *Na*-AIP-1, as there are no experimental structures available for the these two proteins. We identified a small helical region, conserved amongst these hookworm proteins, which can alleviate symptoms in a chemically induced mouse model of colitis and suppress cytokine secretion by human peripheral blood mononuclear cells *in vitro*.

## Materials and methods

### Peptide synthesis and purification

Peptides were synthesized using solid-phase peptide synthesis (SPPS) on a Protein Technologies PS3 synthesizer using fluorenylmethyloxycarbonyl (Fmoc) chemistry on a 0.1 mmol scale. The peptides were synthesized on 2-chlorotrityl chloride resin. Amino acids (2 equiv.) were activated in 0.5 M 2-(1H-benzotriazol-1-yl)-1,1,3,3-tetramethyluronium hexafluorophosphate with 10 equiv. dimethylformamide. The first amino acid was coupled manually to the resin. Following the complete assembly of the peptides, all peptides were cleaved from the resin using a mixture of 95% trifluoroacetic acid (TFA)/2.5% water/2.5% triisopropylsilane for 2–3 h, and then each peptide was precipitated with diethyl ether. After precipitation the peptides were dissolved in 0.05% TFA/50% acetonitrile/50% water, and finally lyophilized. Purification was performed with RP-HPLC on a C_18_ preparative column (Phenomenex Jupiter 250 × 21.2 mm, 10 μm, 300 Å) using a 1% gradient solvent B, (solvent A: 0.05% TFA/100% water; solvent B: 0.05% TFA/90% acetonitrile/10% water). Masses were analyzed using MALDI-TOF mass spectrometry. All compounds were > 95% pure by RP-HPLC.

### Nuclear magnetic resonance spectroscopy and structural analysis

Lyophilized and purified peptides were resuspended to a final concentration of ∼0.2 mM in 90%H_2_O:10%D_2_O, pH 4.5. 2D^1^H-^1^H TOCSY, ^1^H-^1^H NOESY, ^1^H-^1^H DQF-COSY, ^1^H-^15^N HSQC, and ^1^H-^13^C HSQC spectra were acquired at 290 K using a 600 MHz AVANCE III Nuclear magnetic resonance (NMR) spectrometer (Bruker, Karlsruhe, Germany). NOESY spectra were acquired with mixing times of 200–300 ms, and TOCSY spectra were acquired with isotropic mixing periods of 80 ms. Standard Bruker pulse sequences were used with an excitation sculpting scheme for solvent suppression. Spectra were referenced to internal 4,4-dimethyl-4-silapentane-1-sulfonic acid (DSS). For D_2_O exchange experiments peptides were dissolved in 100% D_2_O and 1D and TOCSY spectra recorded over time to monitor the disappearance of amide protons.

The assignments were made using established protocols (26) and the secondary shifts derived by subtracting the random coil αH shift from the experimental αH shifts (27). The 2D NOESY spectra of AIP2-20 were automatically assigned and an ensemble of structures calculated using CYANA (28). Torsion-angle restraints from TALOS-N (29) were used in the structure calculations. The final structures were visualized using MOLMOL (30). The structures of AIP2-20 were deposited to the PDB (PDB code 7T6G) and the BMRB (ID code 30975).

### Trinitrobenzenesulfonic acid colitis model

Experiments were conducted in accordance with the James Cook University Animal Ethics Committee approved guidelines under project #A2012. All experiments were performed with C57BL/6 strain mice in groups of five males (5 weeks old). Mice were purchased from the Animal Resources Centre (Perth, Australia) and housed in the animal care facility unit at James Cook University under specific pathogen-free conditions. After arriving at the facility, mice were randomly placed in ventilated caged system (OptiMICE, Animal Care Systems) inside cages with unlimited access to food and water.

Mice received intraperitoneal (i.p.) injections of peptides dissolved in phosphate buffer saline (PBS) at a dose of 1.0 mg/kg, or oral gavage at 5.0 mg/kg (PBS/olive oil emulsion), 5 h prior to administration of Trinitrobenzenesulfonic acid (TNBS). As a negative non-protective vehicle control peptide, SFTI-1 (14-residue naturally occurring peptide) was administered. Peptides were tested in a Pierce™ chromogenic endotoxin quant kit (Thermo Fisher Scientific) prior to administration to ensure the samples were not contaminated with endotoxin. Ketamine/xylazine solution was used to anesthetize the mice prior to administration of TNBS. Mice received 100 μL of 5% (w/v) TNBS solution in 60% ethanol by enema using a 20-gauge soft catheter (Terumo), which was inserted into the colon. Mice were monitored daily for piloerection, motility, stool consistency, body weight, rectal bleeding and decreased motor activity. The effective induction of TNBS-induced colitis was confirmed by weight loss during the course of the experiment due to inflammation and colonic mucosa damage as compared to untreated healthy naive mice. On day 3 of the experiment, the mice were euthanized using gas asphyxiation and assessed for colitis pathology. Colons were harvested, opened longitudinally, washed in sterile PBS and examined under a stereomicroscope (Olympus SZ61, 0.67–4.5x). A macroscopic pathology score was calculated based on the severity (from 0 to 3) for the following lesions: adhesion, bowel wall thickening, mucosal edema, ulceration, and colon length as described previously (11). The experimental timeline is represented in Supplementary Figure 1. All animal experiments were conducted in at least duplicate on different days to ensure reproducibility of the findings. Graphs and statistical analyses were produced using GraphPad Prism version 8 (GraphPad Software Inc.).

### Histological evaluation of colitis

Tissue for histological analysis was fixed in formalin and then transferred to a solution of 70% alcohol. The tissue was embedded in paraffin and sectioned longitudinally for histology at 4 μm thickness. Periodic acid-Schiff (PAS) stain was used to assess goblet cell destruction. Scoring of the images was determined in a blinded fashion following the scoring method of Hong et al. (31). High resolution images were scored as follows: Ulceration: no ulcers = 0; 1 ulcer = 1; 2 ulcers = 2; 3 ulcers = 3; and > 3 ulcers = 4. Infiltration: 0 = no infiltrate, 1 = infiltrate at crypt bases, 2 = infiltrate reaching to muscularis mucosa, 3 = extensive infiltration reaching the muscularis, and 4 = infiltration of the submucosa with edema. Epithelium was scored: 0 = normal morphology, 1 = loss of goblet cells in one area, 2 = loss of goblet cells in more than one area, 3 = loss of crypts in one area, and 4 = loss of crypts in more than one area. Lymphoid follicles: none = 0, 1 = 1, 2 = 2, 3 = 3, > 3 = 4.

### Bioactivity on human immune cells

The human blood for this research was donated by healthy volunteers. Donor material was studied under the guidelines and regulations of James Cook University (JCU; Cairns, Australia; H7010). Written informed consent was obtained from all participants of the study. PBMCs were isolated from whole blood by density gradient centrifugation using Lymphoprep™ medium (STEMCELL™ Technologies, Vancouver, Canada), according to the manufacturer’s instructions. During experiments, cells were maintained in a culture media containing RPMI 1640 without L-glutamine (Gibco Thermo Fisher Scientific, Waltham, MA, United States), 10% heat-inactivated fetal bovine serum (FBS) (Bovogen Biologicals, Christchurch, NZ), 10,000 units/mL of penicillin + 10,000 μg/mL of streptomycin (Thermo Fisher Scientific), and 1X GlutaMAX (Thermo Fisher Scientific).

The induction of cytokine secretion *in vitro* was achieved by using either a cell stimulation cocktail of 50 ng/mL of phorbol 12-myristate 13-acetate (PMA) and 1 μg/mL ionomycin (eBioscience), or 10 ng/mL lipopolysaccharide (LPS; Sigma-Aldrich). Under each condition, cells were treated with 0.1–100 μg/mL of hookworm AIP peptides or remained untreated. The cell culture plates were incubated for 24 h at 37°C and 5% CO_2_. After incubation, the samples were centrifuged at 500 × g for 5 min, and the culture supernatants were collected for cytokine analysis.

### *BD*™ cytometric bead array

Interleukin (IL)-1β, IL-2, IL-6, IL-8 and tumor necrosis factor TNF from PBMC culture supernatant were quantified using a *BD*™ Cytometric Bead Array (CBA) (BD Biosciences). The CBA assays were performed according to the manufacturer’s instruction using a five laser Special Order LSRFortessa™ with HTS (BD Biosciences). Cytokine concentrations (ng/mL) were calculated based on the sample MFI compared to the cytokine standard curves. *BD*™ FCAP Array software version 3.0 was used for data analysis. Graphs and statistical analysis were produced using GraphPad Prism version 8 (GraphPad Software Inc.).

### Serum stability assay

The serum stability of the peptides was tested using human male AB plasma (Sigma-Aldrich) following methods previously described (32). Peptides were tested at each time point in triplicate. Human serum was prepared by centrifugation at 17,000 g for 10 min to remove the lipid component. Supernatant was incubated for 15 min at 37°C prior to the assay. 200 μM stock solution of each peptide was diluted (1:10) in 100% human serum or PBS at 37°C. 30 μL aliquots were taken at 0, 3, 8, and 24 h. The aliquots of serum were denatured by quenching with 30 μL of 3 M urea and incubated for 10 min at 4°C. Serum proteins were precipitated with the addition of 30 μL of 7% trichloroacetic acid (TCA) for 10 min at 4°C. PBS received the same treatment as serum. Subsequently, the samples were centrifuged at 17,000 g for 10 min. 90 μL of supernatant was analyzed by RP-HPLC at a flow rate of 0.3 mL/min using a Phenomenex Jupiter Proteo C12 analytical column (150 × 2.00 mm, 4 μm, 90 Å). A linear 1% min^–1^ acetonitrile gradient (0–50%) was used for the analysis. The elution time for each peptide was determined by the PBS control for that time point. The absorbance of the eluent was observed using a dual wavelength UV detector set to 214 and 280 nm. The stability at each time point was calculated as the amplitude/area of the serum treated peptide peak on RP-HPLC at 214 nm as percentage of the amplitude/area of the 0 h PBS treated control peptides.

## Results

### Molecular models and peptide design

The structure of *Ac*-AIP-2 was modeled using I-TASSER protein structure and function prediction software, which involves: threading template identification, iterative structure assembly simulation, model selection and refinement, and structure-based function annotation (33). The template with the highest scores was the human TIMP-2 protein (PDB code 1BR9.pdb) (34) highlighting the similarities between these netrin domain-containing proteins.

In an attempt to determine if peptides corresponding to small, discrete elements of secondary structure could maintain the anti-inflammatory activity of *Ac*-AIP-2 (5), we synthesized two peptides comprising residues 7–17 (AIP2-11) and 115–134 (AIP2-20) of *Ac*-AIP-2 which corresponded to α-helices in the modeled structure. In addition to comprising discrete elements of secondary structure, these regions were reasonably solvent exposed in the modeled structure suggesting they might be involved with inter-molecular interactions. The peptides were synthesized using Fmoc chemistry, purified using RP-HPLC and the mass analyzed using MALDI mass spectrometry. Promising results in the TNBS experiments were obtained for the latter peptide, AIP2-20, prompting further study.

**FIGURE 1:**
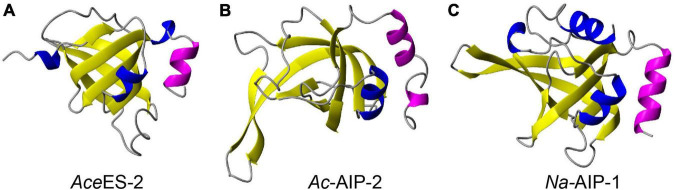
Structures of hookworm proteins. **(A)** Crystal structure of *Ace*ES-2 (PDB code 3NSW). **(B)** The modeled structure of *Ac*-AIP-2. **(C)** The modeled structure of *Na*-AIP-1 (4). The conserved C-terminal helical region is shown in magenta. The extended C-terminal tail in *Ac*-AIP-2 has been removed for clarity. The figure was made using MOLMOL (30).

Comparison of the structure of *Ac*-AIP-2 with the structures of the related hookworm proteins *Ace*ES-2 and *Na*-AIP-1, which also contain netrin domains, is shown in Figure 1. The helical region corresponding to residues 115–134 in *Ac*-AIP-2 is conserved in *Ace*ES-2 and *Na*-AIP-1. The conservation of this helical region is consistent with it being functionally important as helices are often involved in protein-protein interactions. Analysis of protein complexes submitted to the PDB showed that 62% contain a helix at the protein interaction interface (25). Consequently, two additional peptides were designed which corresponded to the C-terminal helical regions in *Ace*ES-2 and *Na*-AIP-1. The sequences of the synthetic peptides are given in Table 1. The peptide names are based on the protein they are derived from and the number of residues. A mutant form of AIP2-20 (AIP2-20D6P), where Asp6 was replaced with a proline residue, was included in the suite of peptides synthesized. The rationale for this peptide was based on the structural data obtained for AIP2-20 (see below).

**TABLE 1:** Sequences of synthetic peptides.

Protein	Residue numbers	Peptide name	Amino acid sequence*
*Ac*-AIP-2	115–134	AIP2-20	TPEEHDLLMDLMGDPKK**A**EE
*Ac*-AIP-2	115–134	AIP2-20D6P	TPEEHPLLMDLMGDPKK***A***EE
*Ac*-AIP-2	7–17	AIP2-11	GTLKEAF***A***QSD
*Na*-AIP-1	125–137	AIP1-13	PSKEKADLGKYK***A***
AceES-2	93–102	ES2-10	SQKEKDLLKE

*The EXXXL motif (underlined) is highly conserved amongst the proteins. **A** Cysteine residues replaced with alanine residues.

### Structural analysis of the synthetic peptides

**FIGURE 2:**
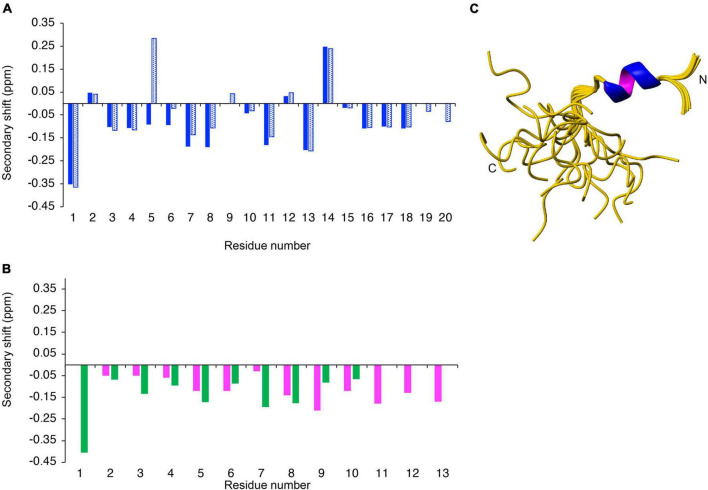
Structural analysis of synthetic peptides. The secondary shifts of **(A)** AIP2-20 (blue) and AIP2-20D6P (blue dotted); and **(B)** AIP1-13 (purple) and ES2-10 (green) were calculated by subtracting the random coil shifts (27) from the αH shift. **(C)** Three-dimensional structure of AIP2-20 (PDB code 7T6G). The 20 lowest energy structures of *Ac*-AIP-2 determined based on NMR spectroscopy data. Superposition of structures over the backbone atoms of residues 3–10 (RMSD 0.172 Å). The structure figure was made using MOLMOL (30).

The structures of the peptides were analyzed in aqueous solution using NMR spectroscopy. The peptides displayed sharp peaks in solution, indicating they were in a monomeric state. Two-dimensional TOCSY and NOESY spectra allowed assignment of the resonances, and the secondary chemical shifts (secondary shifts) were determined by subtracting random coil chemical shifts (27) from the αH chemical shifts. A comparison of the secondary shifts is given in Figures 2A,B. Chemical shift analysis often provides an indication of the type of secondary structure present in peptides, with consecutive shifts more negative than –0.1 signifying the presence of helical structure (35). AIP2-20 displayed consecutive negative chemical shifts in the N-terminal region of the peptide, and AIP1-13 and ES2-10 have consecutive negative secondary shifts for a large proportion of the molecules, albeit with some of the shifts being relatively close to random coil values. The negative shifts present in AIP2-20 are disrupted in AIP2-20D6P at residues 5 and 6. Overall, this analysis indicates a modest propensity for helical structure in the isolated peptides with the exception of AIP2-20D6P where the disruption of negative shifts indicates that the helical region would also be disrupted.

To determine if helical structure is actually present in the peptides, analysis of the NOESY spectra, dihedral angle prediction using TALOS-N (29), and determination of the slowly exchanging amide protons was carried out. Analysis of the NOESY spectra of AIP2-20 indicated the presence of several NOEs (nuclear Overhauser enhancement) indicative of helical turns and several slowly exchanging amide protons were identified in D_2_O exchange experiments. The three-dimensional structures of AIP2-20 were calculated using CYANA, initially based on the NOESY data and dihedral angle restraints predicted using TALOS-N. Based on the preliminary structures and slowly exchanging amide protons, restraints for two hydrogen bonds were included (i,i + 4 CO-NH bonds between Glu4 and Leu8, and His5 and Met9). The final ensemble of AIP2-20 structures is shown in Figure 2C. The structure statistics are given in Supplementary Table 1. The N-terminal region displays a well-defined α-helical region, in contrast to the C-terminal region.

Proline residues generally have a low propensity for helix formation and can result in disruption of helical secondary structure. A proline residue was introduced into the helical region of AIP2-20 to determine if this change disrupted the structure and influenced the activity. The NOESY spectra of AIP2-20D6P showed limited medium or long-range NOEs, and the TALOS-N analysis did not provide any definitive prediction for helical structure, indicating that the D6P mutation disrupts the structure. Slowly exchanging amide protons were evident in the D_2_O exchange experiments but the preliminary structures did not support the presence of hydrogen bonds.

Analysis of the NMR data for the peptides AIP1-13 and ES2-10 demonstrated that both peptides had limited medium or long-range NOEs. TALOS-N analysis did not provide any definitive prediction of the dihedral angles. The lack of medium and long-range cross-peaks in the NOESY spectra and dihedral angle restraints prevented the determination of the structures of AIP1-13 and ES2-10 and suggested that the peptides are not well structured in solution. The peptides displayed slowly exchanging amide protons in the D_2_O exchange experiments but the preliminary structures did not support the presence of hydrogen bonds.

### TNBS-induced weight loss, macroscopic pathology and colon shortening

**FIGURE 3:**
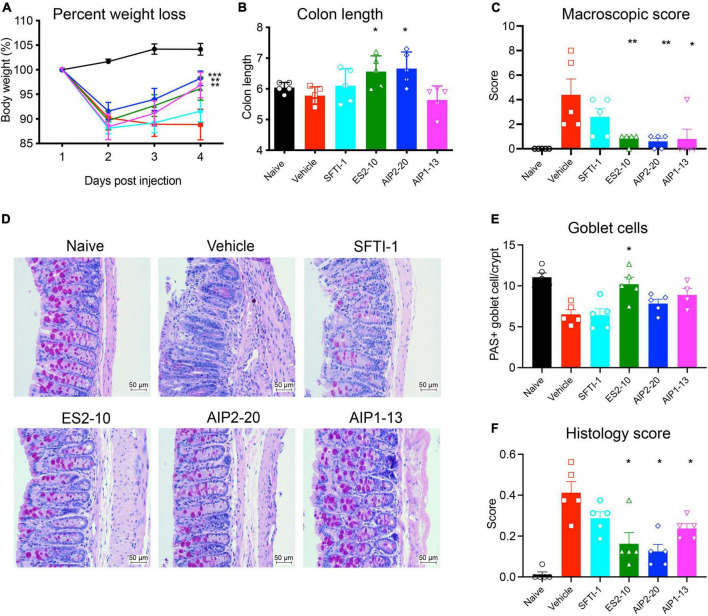
Protective effects of the peptides against weight loss and clinical symptoms induced by TNBS colitis. Mice were untreated (naïve) or treated with TNBS following i.p. administration of peptides, or saline vehicle control (TNBS). **(A)** Body weight percentage; Naive (black); Vehicle (red); SFTI-1 (cyan); ES2-10 (green); AIP2-20 (blue); AIP1-13 (magenta) **(B)** colon length. **(C)** Macroscopic score. **(D)** Representative microscopy of PAS-stained colonic tissue sections. **(E)** Goblet cell scoring. **(F)** Histology scoring. Statistical analyses were performed using GraphPad Prism 8 (2-way ANOVA and unpaired non-parametric Mann-Whitney *t*-test) by comparing to the vehicle-treated colitis group. *P ≤ 0.05; **P ≤ 0.01. All results reported represent means ± standard errors of the means (SEM). There were 5 mice per group, and the experiment was repeated three times.

To test whether any of the peptides display activity in a model of inflammation, we employed the well-characterized TNBS model of colitis. AIP2-20, AIP1-13, and ES2-10 administered with an i.p dose displayed significant protective effects against TNBS-induced intestinal inflammation, as shown in Figure 3. AIP2-20 (*P* < 0.001), AIP1-13 (*P* < 0.01) and ES2-10 (*P* < 0.01) protected against TNBS-induced weight loss (Figure 3A) compared to the vehicle only group (TNBS) and the control peptide SFTI-1 group which, consistent with previous studies, showed no protective effect (36). Treatment of mice with ES2-10 or AIP2-20 resulted in significant protection (*P* < 0.05) against TNBS-induced colon shortening (Figure 3B) compared to TNBS mice, whereas SFTI-1 was not statistically different to the vehicle treated mice. The macroscopic scores for mice treated with all three peptides were significantly lower than the TNBS-only treated mice (Figure 3C). AIP2-11 was also tested in the TNBS model and did not display protective effects (Supplementary Figure 2), indicating that not all regions of the AIP proteins display activity in the colitis model.

Treatment of mice with AIP2-20D6P did not confer protection against any of the parameters measured of TNBS-induced colitis (as shown in Supplementary Figure 3), possibly indicating that Asp6 is important for bioactivity. Alternatively, the structural changes observed as a consequence of this mutation could be responsible for the lack of bioactivity in the mouse model.

### Histological evaluation of colitis

Inflammatory cell infiltration into the lamina propria was visible in the Periodic acid–Schiff (PAS) stain of the colon tissue from the vehicle treated mice (Figure 3D). Colons from vehicle treated mice displayed lesions and histological damage. This tissue exhibited epithelial hyperplasia, goblet cell loss, thickening of the lamina propria and colon walls with extensive ulcerations. By contrast, the mice treated with AIP2-20, AIP1-13, and ES2-10 showed decreased symptoms of colitis, with intact epithelium with minimal focal inflammatory cell infiltrates in the mucosa, no ulceration (Figure 3D) and more abundant goblet cells (Figure 3E). Histological scoring showed that AIP2-20, AIP1-13, and ES2-10 have overall reduced pathology compared with vehicle treated mice (Figure 3F).

### Effects on primary human lymphocytes

**FIGURE 4:**
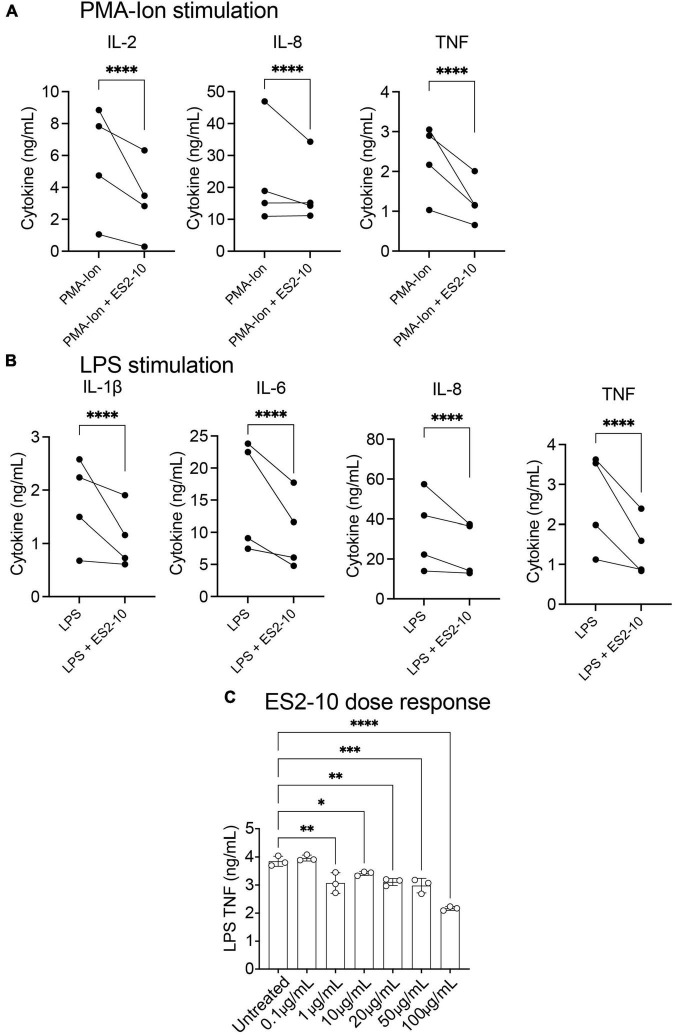
ES2-10 shows suppressive activity on human cytokine secretion. ES2-10 (100 μg/mL) was added to 1 × 10^6^ PBMC from four genetically unrelated donors and stimulated with **(A)** 50 ng/mL PMA and 1 μg/mL ionomycin or **(B)** 10 ng/mL LPS. After 24 h incubation, cytokines from culture supernatants were quantified by CBA. **(C)** ES2-10 dose-response (0.1–100 μg/mL) against LPS-stimulated TNF. All results were performed in triplicate. *P < 0.05; **P < 0.01; ***P < 0.001; ****P < 0.0001. Data are expressed as mean ± standard deviation (SD).

To assess the cytokine suppressive activity of AIP2-20, AIP1-13, and ES2-10 on human immune cells, PBMCs were stimulated with PMA/ionomycin or LPS and treated with peptides for 24 h. Among them, only ES2-10 showed effects in initial experiments (Supplementary Figure 4), and therefore additional assays were carried out using this peptide. ES2-10 treatment significantly decreased the secretion of IL-2, IL-8 and TNF from PMA/ionomycin stimulated-PBMCs from four human donors (Figure 4A). In addition, ES2-10-treated PBMCs released significantly lower levels of LPS-induced IL-1β, IL-6, IL-8 and TNF, compared to control cells (Figure 4B). A peptide dose-response against LPS-stimulated cells revealed TNF inhibition to 1 μg/mL ES2-10 (Figure 4C).

### ES2-10 oral activity

**FIGURE 5:**
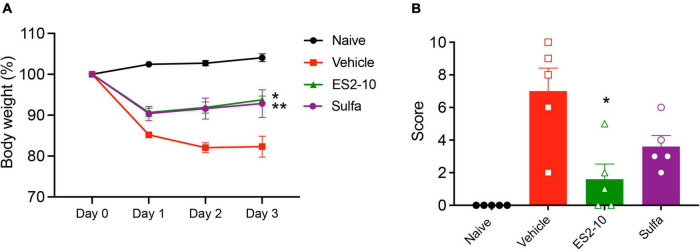
Protective effects of orally administered ES2-10 against weight loss **(A)** and clinical scores **(B)** induced by TNBS colitis. Mice were untreated (naïve) or treated with TNBS following oral administration (5 mg/kg) of ES2-10 or sulfasalazine. Statistical analyses were performed using GraphPad Prism 8 (2-way ANOVA and unpaired non-parametric Mann-Whitney *t*-test). *P ≤ 0.05; **P ≤ 0.01. All results reported represent means ± standard errors of the means (SEM). There were 5 mice per group, and the experiment was repeated three times.

Following the promising activity of ES2-10 with the human cells, we tested the oral activity of the peptide in the TNBS mouse model. A comparison of the oral activity (administered at a dose of 5 mg/kg) of ES2-10 with sulfasalazine is shown in Figures 5A,B. ES2-10 has comparable oral activity to sulfasalazine, a small molecule currently used in the treatment of IBD, based on the weight loss and clinical scores. Neither ES2-10 nor sulfasalazine were distinct from the vehicle treated mice in terms of colon length (Supplementary Figure 5). Comparison of ES2-10 with the *Ac*-AIP-2 protein is shown in Supplementary Figure 6, highlighting the higher potency of the peptide compared to an AIP protein.

### Serum stability assay

**FIGURE 6:**
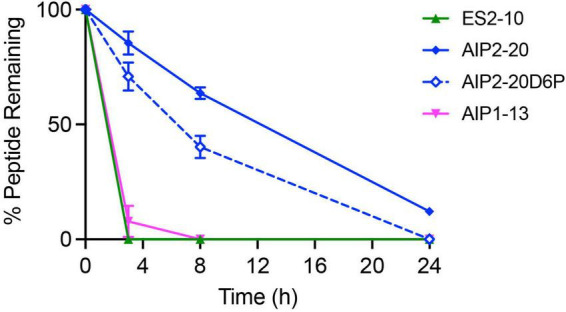
*In vitro* stability of the peptides in human serum. The percentage of peptide remaining in the serum stability assay was assessed by RP-HPLC. Peptides were tested at a concentration of 200 μM, incubated in serum or PBS at 37°C and 40 μL aliquots taken at 0, 3, 8, and 24 h. All data are represented as mean ± SD and were recorded in triplicate.

The stability of the peptides in human serum was assessed over 24 h. The percentage of peptide remaining was determined using RP-HPLC, and the degradation profiles are shown in Figure 6. AIP2-20 degraded slower than AIP1-13 and ES2-10. The latter two peptides had less than 10% peptide remaining within 4 h, whereas AIP2-20 had more than 50% remaining at the 8-h time-point and could still be detected after 24 h. AIP2-20D6P degraded slower than AIP1-13 and ES2-10 but was less stable than AIP2-20.

## Discussion

This study used hookworm AIP proteins to develop new drug leads for inflammatory disease. We have applied a downsizing approach to *Ace*ES-2, *Ac*-AIP-2, and *Na*-AIP-1 to derive peptides of 20 residues or less, with significant protective effects in a mouse model of colitis. In addition, the *Ace*ES-2 derived-peptide suppressed inflammatory cytokines when incubated with human PBMCs *ex vivo*. Our results indicate that a conserved helical region in the proteins is responsible, at least in part, for the anti-inflammatory effects observed in this family of hookworm proteins. Importantly, the *Ace*ES-2 derived peptide displayed oral activity in TNBS colitis, giving this peptide significant potential in drug design.

Comparison of the structures of the active peptides highlighted some intriguing differences. The structure of AIP2-20 contains a well-defined helix over residues 3–10, consistent with the modeled structure of the *Ac*-AIP-2 protein and experimental structure of *Ace*ES-2. Furthermore, the presence of slowly exchanging amide protons is consistent with the presence of hydrogen bonds stabilizing the structure. In contrast, we were unable to determine the structures of ES2-10 and AIP1-13 because of a lack of distance and dihedral angle restraints. ES2-10 and AIP1-13 are considerably shorter than AIP2-20, which can impact the quality of the NOESY spectra, given the relationships between molecular weight, NOE intensity and mixing time (37). However, in this case, strong peaks corresponding to the sequential connections are present, suggesting that the lack of medium and long-range NOEs was not simply a function of the mixing time.

Despite the lack of medium and long-range NOEs for ES2-10 and AIP1-13, these peptides have similar secondary shifts to AIP2-20, with a proportion of negative shifts indicating the presence of helical structure. The presence of slowly exchanging amide protons in ES2-10 and AIP1-13 are indicative of hydrogen bonds stabilizing the structure. Therefore, based on the chemical shifts and slowly exchanging amide protons, it would be expected that AIP1-13 and ES2-10 would also display well-defined helical structures in solution, but this is not the case as the structures could not be defined.

The apparent discrepancy between NOEs, and chemical shifts, and slowly exchanging amide protons has been previously observed in cytochrome b_562_ (38). Similar to our results, cytochrome b_562_ showed chemical shifts and slowly exchanging amide protons consistent with the presence of helical structure, but was not supported by the NOE data. Relaxation analysis indicated the presence of conformational exchange as the likely reason for the discrepancy. It is possible that similar conformational exchange could be occurring with ES2-10 and AIP1-13, but further study is required to confirm this suggestion.

The serum stability results also highlight a difference between the peptides. AIP2-20 is more stable than AIP1-13 and ES2-10. The reasonably high stability of AIP2-20 is consistent with the well-defined helical structure present in solution, whereas degradation of ES2-10 and AIP1-13 is consistent with a lack of secondary structure. It is unclear how conformational exchange in ES2-10 and AIP1-13 could impact the serum stability, but overall, it appears that the longer peptide (AIP2-20) has the most stable structure. AIP2-20D6P has improved stability in serum compared to AIP1-13 and ES2-10 but is less stable than AIP2-20, indicating that the introduction of a proline residue in the helical region decreases the biological stability.

A conserved sequence motif (EXXXL) is present in the peptides derived from the three AIP proteins studied (Table 1). A hydrogen bond between the conserved glutamic acid and leucine residues is present in the AIP2-20 structure, the crystal structure of the *Ace*ES-2 protein, and the modeled structures of *Ac*-AIP-2 and *Na*-AIP-1. Consistent with the formation of a hydrogen bond, the leucine residue is slowly exchanging in AIP2-20, as well as AIP1-13 and ES2-10, despite the latter two peptides not displaying well-defined structures in solution. It is possible that this sequence motif, with a propensity to form a hydrogen bond, is important for bioactivity. However, only ES2-10 suppressed cytokines in human PBMCs suggesting that the sequence diversity is responsible for the differences observed with the human cells. Given the activity displayed by ES2-10 in human PBMC assays, we further explored the potential of this peptide by testing its oral bioactivity in a TNBS mouse colitis model. Interestingly, this peptide displayed relatively potent activity (Figure 5), highlighting its potential as a drug lead. The colitis model used in the current study involved pre-treatment of the peptide prior to administration of TNBS. It will be of interest to test the peptides in a model with pre-existing inflammation to determine their effectiveness in a more clinical setting.

Previous studies on hookworm peptides and proteins has provided some insight into mechanisms of action. For instance, some netrin domain-containing proteins, including TIMPs, have biological functions unrelated to MMP inhibition, including inhibition of cell migration (39). Depletion of either CD11c^+^ dendritic cells or Foxp3^+^ regulatory T cells ablated AIP-2-induced protection against asthma (5) and *Na*-AIP-1-induced protection against colitis (4). Furthermore, synthetic, disulfide-rich peptides based on ES products of *A. caninum* and *N. americanus*, reduced colitis symptoms in a TNBS model and suppressed CD4 + T cell proliferation and inhibited IL-2 and TNF production (40). The effects that we detected with ES2-10 on human PBMCs indicated that the peptide suppressed cytokine production during LPS-stimulation, which primarily involves myeloid cells such as monocytes. However, we also detected ES2-10-induced suppression of cytokine production after stimulation of PBMCs with PMA-ionomycin, which is a potent stimulus for T cell cytokine production. Whether ES2-10 acts to suppress human cytokine responses *via* myeloid cells, or lymphoid cells such as T cells remains unclear.

In summary, a small helical region present in hookworm netrin domain-containing Anti-Inflammatory Proteins (AIPs) appears to play a role in their bioactivity. This region is relatively solvent exposed in the hookworm proteins and therefore has the potential to be involved in protein-protein interactions. Consequently, we have identified a promising starting point for the design of peptide-based lead molecules to treat inflammatory diseases such as IBD but they might also be applicable to other chronic inflammatory disease such as asthma. Further research into other proteins and peptides that worms secrete may reveal additional therapeutic products that could be used in combination with the ES2-10 peptide, to more closely mimic the myriad of immunomodulatory functions that live worms release into their host.

## Data availability statement

The datasets presented in this study can be found in online repositories. The names of the repository/repositories and accession number(s) can be found in the article/Supplementary material.

## Ethics statement

The studies involving human participants were reviewed and approved by the James Cook University Human Ethics Committee. The patients/participants provided their written informed consent to participate in this study. The animal study was reviewed and approved by the James Cook University Animal Ethics Committee.

## Author contributions

ND, AL, and PB: conceptualization. CC, ND, DW, PB, DP, MF, JM, RR, PG, and SN: methodology. CC, ND, DW, PB, DP, LJ, GZ, RE, RYMR, CNR, RR, PG, and SN: investigation. ND, DW, and AL: supervision. CC and ND: writing—original draft. CC, ND, DW, SN, PG, RR, JM, RYMR, and AL: writing—review and editing. All authors contributed to the article and approved the submitted version.
