# Supplementary material for: Peptides derived from hookworm anti-inflammatory proteins suppress inducible colitis in mice and inflammatory cytokine production by human cells
Source: Front Med (Lausanne). 2022 Sep 9;9:934852. doi: 10.3389/fmed.2022.934852 (PMC9524151; doi:10.3389/fmed.2022.934852)
Supplement: Supplementary file 1 [file Data_Sheet_1.pdf]

Supplementary Data

Peptides derived from hookworm anti-inflammatory proteins suppress inducible colitis in mice and inflammatory cytokine production by human cells

Claudia Cobos, Paramjit S. Bansal, David T. Wilson, Linda Jones, Guangzu Zhao, Matthew A. Field, Ramon M. Eichenberger, Darren A. Pickering, Rachael Y. M. Ryan, Champa N. Ratnatunga, John J. Miles, Roland Ruscher, Paul R. Giacomin, Severine Navarro, Alex Loukas[*], Norelle L. Daly[*]

Centre for Molecular Therapeutics, Australian Institute of Tropical Health and Medicine, James Cook University, Cairns, QLD Australia.

[*]To whom correspondence should be addressed:

Professor Norelle L. Daly
Address: Centre for Molecular Therapeutics, AITHM, James Cook University, QLD 4870, Australia
Email: norelle.daly@jcu.edu.au
Phone: +61-7-4232 1815

Professor Alex Loukas
Address: Centre for Molecular Therapeutics, AITHM, James Cook University, QLD 4870, Australia
Email: alex.loukas@jcu.edu.au
Phone: +61-7-4232 1608

Supplementary Figure 1

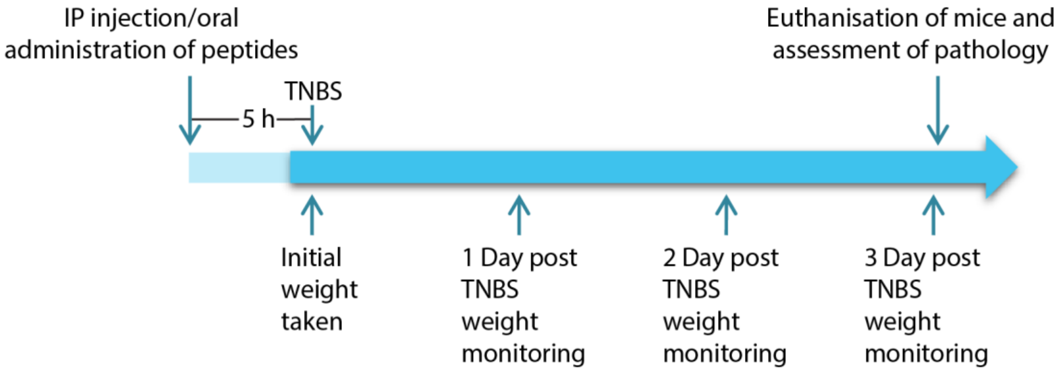

Supplementary Figure 1: Graphical representation of the experimental set up for the TNBS mice studies. The peptides or controls were administered 5 hours prior to administration of TNBS. The weight of the mice was monitored on the day of TNBS administration and then subsequently for three days, prior to euthanising the mice and assessment of the pathology.

Supplementary Figure 2

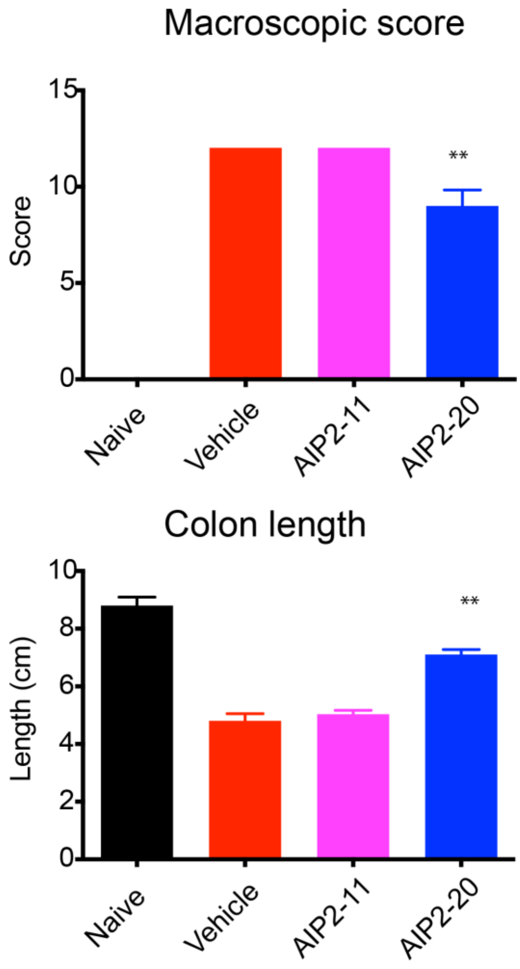

Supplementary Figure 2: **Effects of *Ac*-AIP-2 derived peptides on macroscopic pathology and colon shortening induced by TNBS colitis.** Mice were untreated (naïve) or treated with TNBS following i.p. administration of peptide, or saline vehicle control (TNBS). Statistical analyses were performed using GraphPad Prism 8 (unpaired nonparametric Mann-Whitney t-test). *P ≤ 0.05; **P ≤ 0.01; ***P ≤ 0.001; ****P ≤ 0.0001. All results reported represent means ± standard errors of the means (SEM). There were 5 mice per group. The peptides tested corresponded to residues 7-17 (AIP2-11) and 115-134 (AIP2-20) of *Ac*-AIP-2.

Supplementary Figure 3

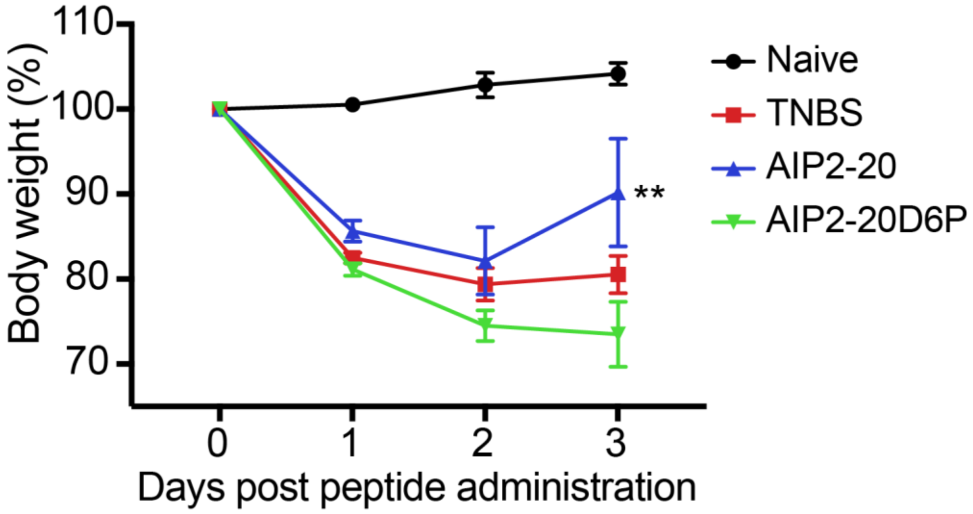

Supplementary Figure 3: Percent change in body weight following treatment with AIP2-20 and AIP2-20D6P in a TNBS colitis model. Mice were untreated (naïve) or treated with TNBS following i.p. administration of peptides (1mg/kg), or saline vehicle control (TNBS). AIP2-20D6P did not protect against the symptoms of colitis in contrast to AIP2-20. ** $P < 0.01$.

Supplementary Figure 4

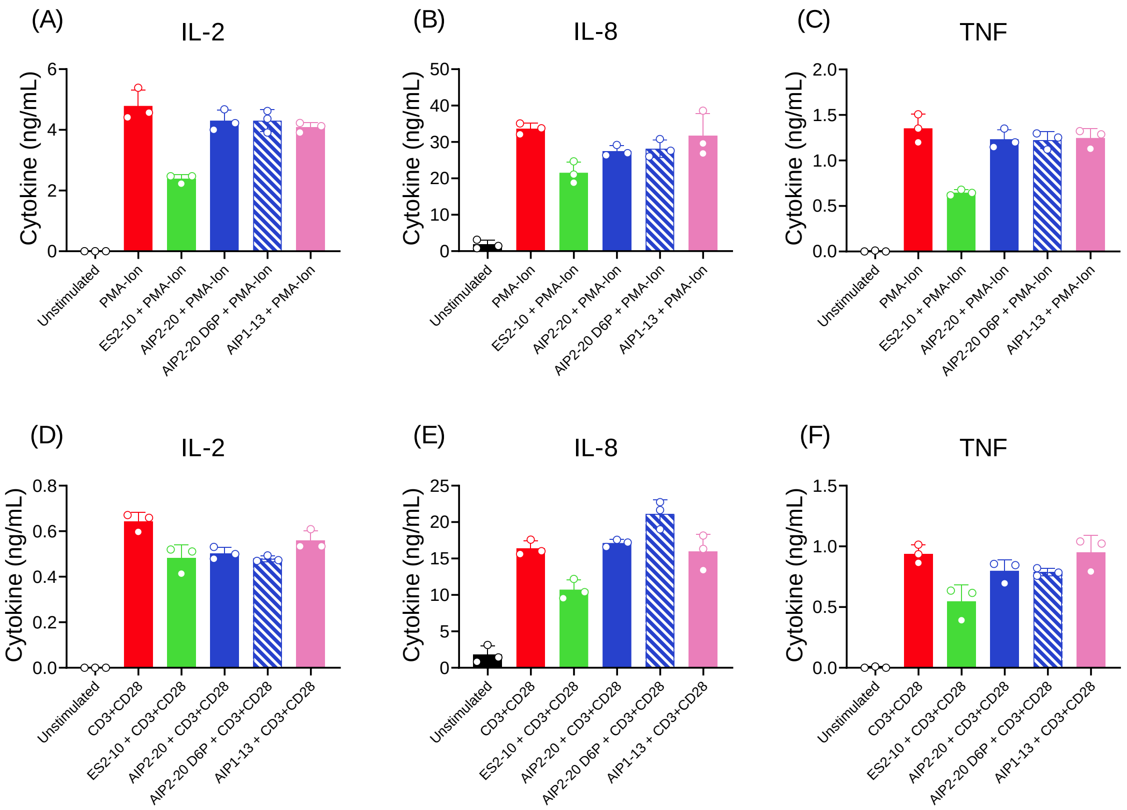

Supplementary Figure 4: Initial screen for peptide-induced suppression of T cell cytokine secretion. Human PBMCs ($1.3x10^5$/100 µL medium) were stimulated with **(A-C)** 50 ng/mL phorbol 12-myristate 13-acetate + 1 µg/mL ionomycin (PMA-Ion) or **(D-F)** Dynabeads Human T-Activator beads (CD3+CD28) at a ratio of 1:1 (bead to cell). The activated cells were treated with 100 µg/mL AIP peptides for 24 h. Secreted IL-2, IL-8, and TNF were quantified by cytometric bead array. Bars show the mean cytokine concentration (ng/mL) ± SD of triplicate samples (*n* = 1 donor).

Supplementary Figure 5

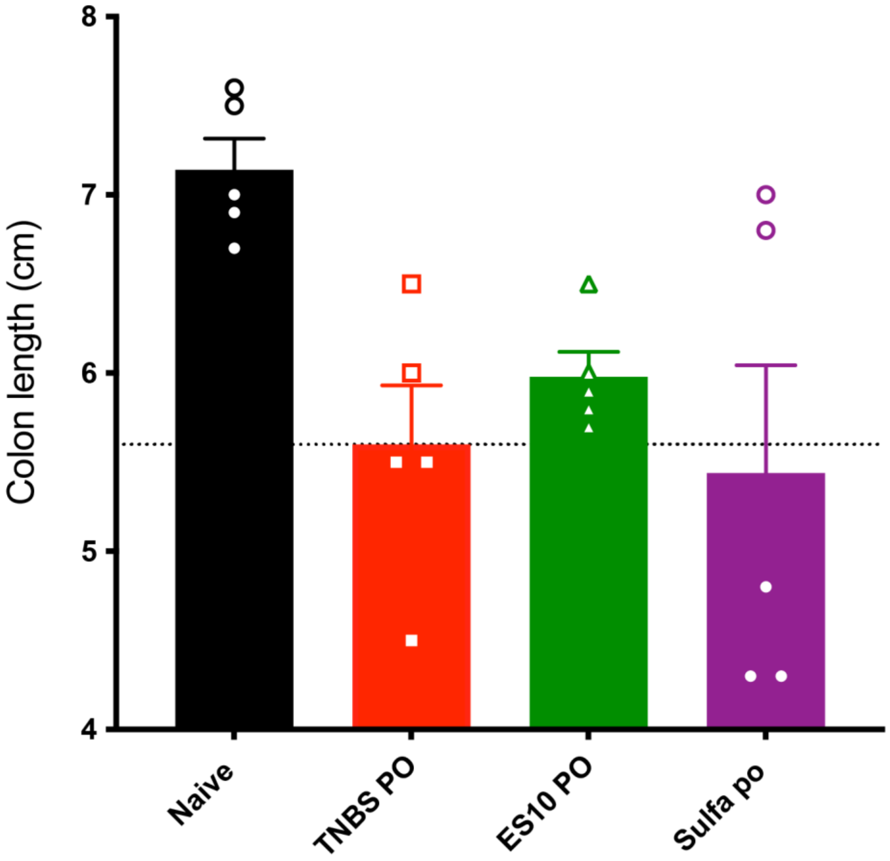

Effects of orally administered ES2-10 on colon length. Mice were untreated (naïve) or treated with TNBS following oral administration (5 mg/kg) of ES2-10 or sulfasalazine. There were 5 mice per group, and the experiment was repeated three times.

Supplementary Figure 6

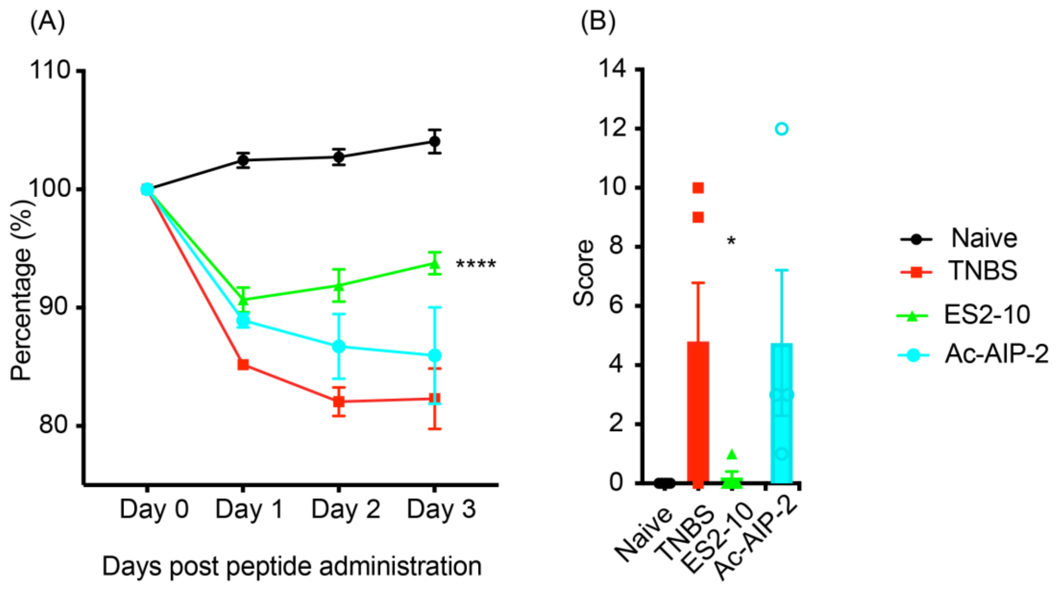

Supplementary Figure 5: **Effects of an ES2-10 and *Ac*-AIP-2 on weight loss (A) and macroscopic pathology (B) induced by TNBS colitis.** Mice were untreated (naïve) or treated with TNBS following oral administration of ES2-10 or *Ac*-AIP-2 (formulated in PBS/olive oil 50% v/v), or PBS/olive oil vehicle control (TNBS). Statistical analyses were performed using GraphPad Prism 8 (unpaired nonparametric Mann-Whitney t-test). *P ≤ 0.05; **P ≤ 0.01; ***P ≤ 0.001; ****P ≤ 0.0001. All results reported represent means ± standard errors of the means (SEM). There were 5 mice per group.

Supplementary Table S1

Structural statistics for the truncated Ac-AIP-2 ensemble	
**Experimental restraints**	
Interproton distance restraints	177
Intraresidue	60
Sequential	86
Medium range (i-j < 5)	31
Dihedral-angle restraints	12
**R.m.s. deviations from mean coordinate structure (Å)**	
Backbone atoms (residue 3-10)	0.24 ± 0.09
All heavy atoms (residue 3-10)	1.17 ± 0.31
**Ramachandran Statistics**	
% in most favoured region	70.7%
% in additionally allowed region	29.3%
